# Unlocking the potential of sacubitril/valsartan therapy in improving ECG and echocardiographic parameters in heart failure patients with reduced ejection fraction (HErEF)

**DOI:** 10.1186/s43044-024-00468-4

**Published:** 2024-03-28

**Authors:** Lamyaa Elsayed Allam, Ahmed Aly Abdelmotteleb, Hayam Mohamed Eldamanhoury, Hassan Shehata Hassan

**Affiliations:** Department of Cardiology, Faculty of Medicine, Ain Shams University, 48 Mohammed Elnadi Street, 6th Zone, Nasr City, Cairo 11371 Egypt

**Keywords:** Sacubitril/valsartan combination, ECG, Echocardiography, Heart failure, Reduced ejection fraction

## Abstract

**Background:**

Sacubitril/valsartan therapy has been found to reduce hospitalizations, improve echocardiogram parameters, and improve mortality in HFrEF. The objective is to assess S/V therapy effect on electrocardiogram indices and how those parameters related to echocardiographic parameters.

**Results:**

From June 2022 until June 2023, this prospective study enrolled 100 patients (mean age 56.1, 8.2, 78% male) with non-ischemic dilated cardiomyopathy (NIDCM) used PARADIGM-HF criteria: NYHA Class II, III, or IV HF; ejection fraction EF ≤ 40%; and hospitalization for HF within previous 12 months. Before starting S/V therapy, an echo and ECG were performed, as well as 6 months following the optimal dose and if LVEF was improved by more than 5%, they were termed notable S/V treatment responders. Aside from improving echo parameters, ECG parameters improved significantly. The QRS width was reduced from 123.7 ± 20.3 to 117.1 ± 18.8 ms (*p* 0.00), and QTc interval was reduced from 425.4 ± 32.8 to 421.4 ± 32.3 ms (*p* = 0.012). QRS width was significantly reduced in patients with LBBB, RBBB, and IVCD based on QRS morphology. QRS width (*r* = − 0.243, *p* = 0.016) and QTc (*r* = − 0.252, *p* = 0.012) had a negative connection with LVEF.

**Conclusion:**

S/V therapy, in addition to improving echo parameters and NYHA class, improves QRS width and corrected QTc interval on ECG in HFrEF patients. This is an indication of reverse electrical LV remodeling and can be used as an auxiliary prediction for tracking therapy outcomes.

## Background

Heart failure is a widely dispersed clinical illness that has significant social and economic implications around the world.

In comparison to Enalapril, the sacubitril/valsartan (S/V), with neprilysin inhibitor/angiotensin II receptor blocker (ARB) combination demonstrated a 20% reduction in heart failure (HF) hospitalizations and cardiovascular deaths in PARADIGM-HF trial [1].

Since publication of this trial in 2014 and FDA approval in 2015 and HF guideline amendment in 2017 [2], (S/V) therapy has become a class I recommendation as a replacement for angiotensin-converting enzyme inhibitors (ACEI) For ambulatory individuals with HFrEF in the recent guidelines [2, 3].

Additionally, a subanalysis of the PARADIGM-HF trial revealed also S/V therapy reduced sudden cardiac death by 20%, with no difference between patients with or without an implantable cardioverter defibrillator (ICD) [1].

Its further effects on tissue remodeling are being investigated in various trials, which explored the potent additional reversal structural remodeling impact of sacubitril/valsartan detected by echocardiography parameters and found that S/V therapy significantly improves LV systolic remodeling and functional mitral regutgitation [4, 5].

Electrical remodeling and ventricular Dyssynchrony can cause disturbances and progression of dyssynergic wall motion, leading to impaired contractile function and heart failure. This condition has been extensively investigated, especially in the left bundle branch block (LBBB). Previous studies in animal models of dyssynchronous heart failure (HF) have documented alterations in calcium ion (Ca^2+^) dynamics, (SERCA and PLB) and gap junction remodeling, especially in the late-activated, high-stress LV free wall that could partly explain the LV function deterioration and propensity to arrhythmias [6–9].

S/V therapy was also associated with a significant decrease in non-sustained and sustained ventricular tachycardia episodes, appropriate ICD shocks, premature ventricular contractions, and, as a result, an increase in biventricular pacing percentage [10, 11].

However, the exact mechanism by which S/V therapy reduces ventricular arrhythmias is unclear. This antiarrhythmic action has been related to several potential mechanisms. It is unclear whether this reduction is due to reversal remodeling, a decrease in cardiac fibrosis, wall stretch, or sympathetic nervous system activation or even reversal electrical remodeling [12–14]

Although electrocardiographic (ECG) changes can provide further information regarding the protective mechanisms associated with sacubitril/valsartan therapy, there are few data on the effect of S/V medication on ECG parameters and most of these studies are retrospective studies.

Based on these findings, the goal of this research was to determine how sacubitril/valsartan (S/V) medication affected electrocardiogram (ECG) parameters in patients with HFrEF, a marker for reverse electrical remodeling, and how those parameters related to echocardiographic parameters.

## Methods

### Patient selection

Patients with HFrEF due to non-ischemic dilated cardiomyopathy (NIDCM) were prospectively enrolled using PARADIGM-HF criteria after permission by Ain-Shams University’s ‘ethics committee’ and written consent from the patients. These criteria included: NYHA Class II, III, or IV heart failure; an ejection fraction (EF) of 40% or less; and being hospitalized for heart failure within the previous 12 months. All these patients had either coronary angiography (CA) or multi-Slice Computed Tomography coronary angiography (MSCT) to exclude coronary artery disease. This study was from June 2022 to June 2023. Patients who had taken a steady dose of any ACE (angiotensin converting enzyme inhibitor) or ARB (angiotensin II receptor blocker) and beta-blocker for at least 4 weeks were invited for participation.

A systolic blood pressure ≤ 100 mmHg, an estimated glomerular filtration rate (eGFR) ≤ 30 mL/min/1.73 m^2^ of body surface area, a serum potassium level ≥ 5.2 mmol/L at assessment, a history of angioedema, or unacceptable adverse effects while taking an ACE inhibitor or an ARB were all exclusion criteria. Other exclusion criteria were HF patients due to organic valvular disease, Hypertrophic cardiomyopathy, arrhythmogenic cardiomyopathy, Histroy of acute coronary syndrome prior to the examination or coronary revascularization, planned revascularization, and patients on antiarrhythmic drugs rather than beta blockers or on paced rhythm either RV pacing or biventricular pacing).

According to previous studies, a 5% or more improvement in LVEF has been considered a significant response to S/V therapy [5].

To lower the risk of angioedema caused by overlapping ACE and neprilysin inhibition, ACE drugs were stopped at least 36 h before beginning S/V therapy. It was not advised to use an ACE inhibitor (or ARB) with S/V therapy. Patients underwent a clinical assessment that included NYHA classification, 12 lead electrocardiography (ECG), trans-thoracic echocardiography (TTE) prior to initiating S/V therapy. The same measurements were repeated six months after establishing S/V combination therapy at the maximum tolerated dose, which was determined by uptitration of the dose based on patient tolerance after blood pressure assessment and also guided by blood investigations such as serum creatinine and potassium levels.

### Electrocardiogram (ECG) measurements

A standard 12-lead ECG was acquired at rest using the Cardiovit AT-102 G2 ECG machine (Schiller, USA), with three limb leads (I, II, and III), three augmented limb leads (aVR, aVL, and aVF), and six precordial leads (V1–V6).

The standard speed and voltage were 25 mm/s and 10 mm/mV, respectively.

Two qualified blinded electrophysiologists recorded ECG measurements such as cycle length, rhythm, PR interval, QT interval, QRS width in milliseconds, QRS morphology, ST segment, and T wave inversion confirmed by machine analysis and then the average measurement for each parameter was documented.

The width of the QRS waves was measured from the onset to the end of the QRS complex in milliseconds. QRS morphology was classified as LBBB in the presence of a broad notched or slurred R wave in leads I, aVL, V5, and V6, as well as the absence of a Q wave in leads I, V5, and V6, and as RBBB in the presence of rsr′, rsR′, or rSR′ complexes in leads V1 or V2. Subjects who did not match these criteria had indeterminate ventricular conduction delay (IVCD) [15].

The QT interval was measured using Tangent method from the start of the Q wave till the end of the T wave and was corrected for heart rate using Bazett’s formula (QTc interval) [15].

### Transthoracic echocardiographic (ECHO) parameters

All patients had routine transthoracic echocardiography with machine integrated electrocardiogram recording utilizing a GE Healthcare Vivid S5 outfitted with a 3 MHZ transducer. An echocardiographer certified by the ECAVI performed a standard study on all subjects using standard techniques [16] to obtain the following measurements: The LV dimensions (LVEDD, LVESD, SWT, and PWT) were obtained using M mode from parasternal short axis view at the level of the papillary muscles; LV ejection fraction (LV EF) was determined using Simpson’s method of diss [16]; and transmittal pulsed-wave Doppler was recorded, with the E/A ratio and E wave deceleration time calculated. The apical four-chamber view was used for offline color-coded tissue Doppler imaging, with the sample volume arranged across the septal and lateral mitral annuli, and early and late diastolic velocities (E′ and A′) were determined. The average E′ velocities at the sepal and lateral mitral annuli, as well as the E/E′ ratio, were calculated. As a result, LV diastolic dysfunction of each patient was assessed in accordance with recommendation [17].

The degree of mitral regurgitation (MR) was quantified using color flow and CW Doppler, and the anteroposterior diameter of the left atrium in the parasternal long axis view was measured using M mode [17].

### Statistical analysis

Data were collected, revised, coded, and entered into the Statistical Package for Social Science (IBM SPSS) version 23. SPSS Inc. products are registered trademarks of SPSS Inc., part of IBM Company, Chicago, USA. When quantitative data were found to be parametric, they presented as mean, standard deviations, and ranges, and median, and interquartile range (IQR) when the data were found to be nonparametric. Qualitative variables were presented as numbers and percentages. The comparison between groups with qualitative data was done using the Chi-square test. The comparison between two paired groups with quantitative data and a parametric distribution was done using a paired t test. The comparison between patients before and after six months from initiation of sacubtril\valsartan therapy with quantitative data and parametric or nonparametric distribution was done by using the chi-square test and paired *t* test. The confidence interval was set to 95%, and the margin of error accepted was set to 5%. So, the *p* value was considered to be the following: *p* > 0.05, non-significant; *p* < 0.05, significant; and *p* < 0.01, highly significant.

## Results

### Demographic data

The research was completed by 100 of 125 patients, with an absolute improvement in LVEF of 5% or more.

Prior to the start of sacubitril/valsartan combination medication, the demographic and clinical data of the study group are shown in Table 1.

**Table 1:** Demographic and clinical data of the study group

Parameter	Result
Age (Yrs): mean ± SD	56.00 ± 8.22
*Gender*	
Male, *n* (%)	78/100 (78%)
Female, *n* (%)	22/100 (22%)
BMI(Kg/m^2^): mean ± SD	24.76 ± 3.48
*Risk factors & comorbidities*	
DM, *n* (%)	56/100 (56%)
Hypertension, *n* (%)	70/100 (70%)
Renal impairment, *n* (%)	18/100 (18%)
Dyslipidemia, *n* (%)	68/100 (68%)
Smoking, *n* (%)	62/100 (13%)
Alcoholic, *n* (%)	2/100 (2%)
OSA, *n* (%)	11/100 (11%)
*Drug treatment*	
β Blockers, *n* (%)	100/100 (100%)
Loop diuretics, *n* (%)	88/100 (88%)
MRAs, *n* (%)	98/100 (98%)
SGLT2 inhibitor, *n* (%)	100/100 (100%)
Empagliflozin	19/100 (19%)
Dapagliflozin	81/100 (81%)
*NYHA class*	
I, *n* (%)	13/100 (13%)
II, *n* (%)	55/100 (55%)
III, *n* (%)	32/100 (32%)
IV, *n* (%)	0
*Echo parameters*	
LV EF (%)	31.59 ± 5.68
LVEDD (cm)	6.38 ± 0.62
LVESD (cm)	5.00 ± 0.83
LAD (cm)	4.59 ± 0.56
DD (≥ II grade)%	63/100 (63%)
Mitral regurgitation (MR) (≥ II grade) %	60/100 (60%)
*ECG parameters*	
Heart rate (beats per minute)	78.14 ± 9.98
Atrial fibrillation (%)	23/100 (23%)
PR interval (ms)	138.53 ± 26.72
QRS width (ms)	123.70 ± 20.32
QTc interval (ms)	425.37 ± 32.86
T wave morphology changes (%)	74/100 (74%)
ST segment changes (%)	60/100 (60%)
*QRS width*	
Wide ≥ 120 ms	69 (69%)
Narrow < 120 ms	13 (13%)
*QRS morphology (in patients with wide QRS width)*	
LBBB	25 (25%)
RBBB	17 (17%)
IVCD	27 (27%)

BMI, body mass index (kilogram/meter^2^); ICM, ischemic cardiomyopathy; NIDCM, non-ischemic dilated cardiomyopathy; DM, diabetes mellitus; OSA, obstructive sleep apnea; MI, myocardial infarction; PCI, percutaneous coronary intervention; CABG, Coronary artery bypass graft; VHD, valvar heart disease; MRAs, Aldosterone receptor antagonists;SGLT2 inhibitor, Sodium-glucose co-transporter 2 inhibitors; NYHA Class, New York Heart association classification; LV EF, left ventricular systolic ejection fraction; LVEDD, left ventricular end diastolic dimension; LVESD, left ventricular end systolic dimension; LAD, Left atrial dimension; DD, LV diastolic dysfunction; QTc interval, corrected QT interval; cm, centimeter; ms, millisecond; LBBB, left bundle branch block; RBBB, right bundle branch block; IVCD, intraventricular conduction delay

### Follow-up after six months

The clinical response of the study patients after six months of sacubitril/valsartan combination (changes in NYHA functional, echocardiographic response, and electrocardiographic response are listed in (Tables 2, 3 and 4).

**Table 2:** NYHA classification of the study population before and after sacubitril/valsartan combination medication commencement

Study population	Before S/V therapy	Six months after S/V therapy	Test value	*p*-value
*NYHA class*				
NYHA class 1	13/100 (13.0%)	30/100 (30.0%)	9.728*	0.008
NYHA class 2	55/100 (55.0%)	50/100 (50.0%)		
NYHA class 3	32/100 (32.0%)	20/100 (20.0%)		

NYHA Class, New York Heart association classification

*Chi-square test

**Table 3:** Echocardiography parameters before and after the sacubitril/valsartan combination was initiated

Before S/V therapy		Six months after S/V therapy	Test value	*p*-value
*LVEF (%)*				
Mean ± SD	31.59 ± 5.68	34.44 ± 7.77	5.256**	0.000
*LVEDD (cm))*				
Mean ± SD	6.38 ± 0.62	6.08 ± 0.63	− 8.657**	0.000
*LVESD (cm)*				
Mean ± SD	5.00 ± 0.83	4.91 ± 0.79	− 2.400**	0.018
*LAD (cm)*				
Mean ± SD	4.59 ± 0.56	4.49 ± 0.48	− 2.882**	0.005
*DD*				
Grade I	21/100 (25.0%)	26/100 (32.9%)	10.188*	0.006
Garde II	36/100 (42.9%)	44/100 (55.7%)		
Garde III	27/100 (32.1%)	9/100 (11.4%)		
*MR*				
No	4/100 (1.0%)	6/100 (6.0%)	6.711*	0.243
Grade I	36/100 (36.0%)	33/100 (33.0%)		
Grade II	54/100 (54.0%)	53/100 (53.0%)		
Garde III/IV	6/100 (6.0%)	3/100 (3.0%)		

LV EF, left ventricular systolic ejection fraction; LVEDD, left ventricular end diastolic dimension; LVESD, left ventricular end systolic dimension; LAD, Left atrial dimension; DD, LV diastolic dysfunction; MR, Mitral regurgitation; cm, centimeter

*Chi-square test; **Paired *t* test

**Table 4:** ECG parameters prior to and following the start of the sacubitril/valsartan combination

	Before S/V therapy	Six months after S/V therapy	Test value	*p*-value
*HR(bpm)*				
Mean ± SD	78.14 ± 9.98	73.05 ± 7.64	− 5.394**	0.000
*Rhythm*				
Sinus	77/100 (77.0%)	71/100 (71.0%)	2.563*	0.278
hy				
AF	23/100 (23.0%)	27/100 (27.0%)		
*PR interval (ms)*				
Mean ± SD	138.53 ± 26.72	137.11 ± 24.20	− 0.541**	0.590
*QRS width*				
Mean ± SD	123.70 ± 20.32	117.05 ± 18.79	− 6.435**	0.000
*QTc*				
Mean ± SD	425.37 ± 32.86	421.35 ± 32.32	− 2.573**	0.012
*T wave morphology*				
Normal	11/100 (11.0%)	11/100 (11.0%)	2.737*	0.603
Inverted T	64/100 (64.0%)	67/100 (67.0%)		
Flat T	10/100 (10.0%)	9/100 (9.0%)		
Biphasic T	15/100 (15.0%)	11/100 (11.0%)		
*ST segment*				
Normal	12 (12.0%)	19 (19.0%)	4.693*	0.196
Depressed ST	73 (73.0%)	69 (69.0%)		
Elevated ST	15 (15.0%)	10 (10.0%)		

HR, heart rate; Bpm, beats per minute; QTc interval, corrected QT interval; ms, millisecond

*Chi-square test; **Paired *t*-test

### NYHA classification (Table 2)

The NYHA functional class improved significantly after S/V combination therapy (*p* 0.008).

### Echo parameters (Table 3)

Several echo parameters improved with sacubitril/valsartan therapy: LVEF increased from 31.59 ± 5.68 to 34.44 ± 7.77% (*p* 0.000); LVEDD decreased from 6.38 ± 0.62 to 6.08 ± 0.63 cm (*p* 0.000); LVESD decreased from 5 ± 0.83 to 4.9 ± 0.79 cm (*p* 0.018); and LAD reduced from 4.59 ± 0.56 to 4.49 ± 0.48 cm (*p* 0.005). In terms of mitral regurgitation, no substantial changes were detected.

### ECG parameters (Table 4)

Mean QRS duration showed significant reduction after S/V therapy, decreasing from 123.70 ± 20.32 to 117.05 ± 18.79 ms (*p* 0.000). QTc interval showed also significant shortening, decreasing from 425.37 ms ± 32.86 to 421.35 ms ± 32.32 ms (*p* 0.012).

### Correlations

**Fig. 1:**
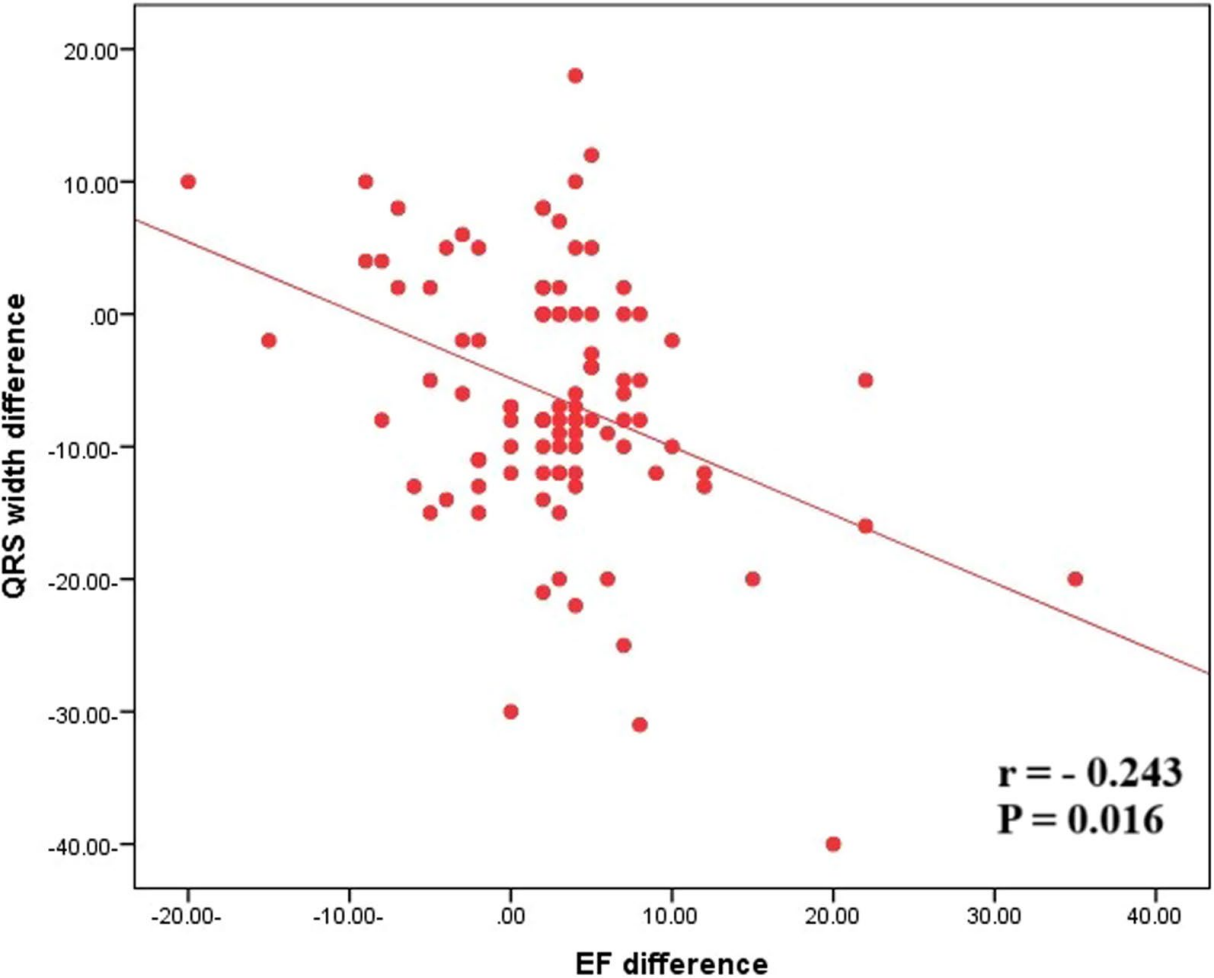
Pearson correlation coefficient (*r*) and *p*-value (*p*) between QRS width change and LV ejection fraction after S/V therapy in HErEF patients

**Fig. 2:**
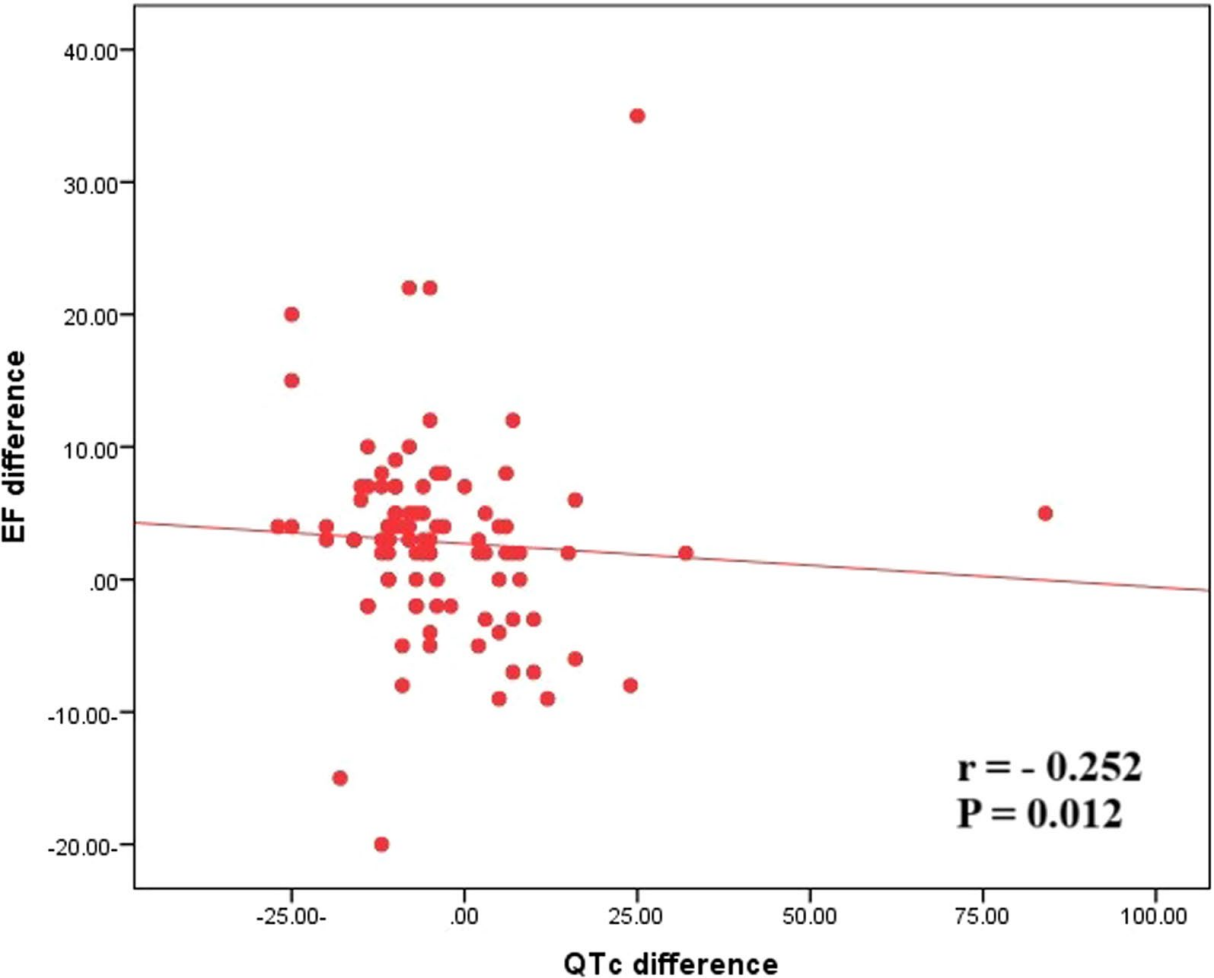
Pearson correlation coefficient (*r*) and *p*-value (*p*) between corrected QT interval change and LV ejection fraction after S/V therapy in HErEF patients

QRS width showed a significant negative correlation with improvement in LVEF (*r *= − 0.243, *p* = 0.016) and QTc interval showed a significant negative correlation with improvement in LVEF (*r *= − 0.252, *p* = 0.012), as shown in Tables 5 and 6 and Figs. 1 and 2.

**Table 5:** Correlation between QRS width change and LV EF (%) after 6 months of S/V therapy

QRS width (ms)	LV EF %	
	*R*	*p*-value
	− 0.243*	0.016

LV EF, left ventricular systolic ejection fraction; ms, millisecond

*Spear Mann correlation coefficient

**Table 6:** Correlation between QTc interval change and LV EF (%) after 6 months of S/V therapy

QTc interval (ms)	LV EF %	
	*R*	*p*-value
	− 0.252*	0.012

QTc interval, corrected QT interval; ms, millisecond

*Spear Mann correlation coefficient

### In patients with a wide QRS (≥ 120 ms) on ECG, see Table 7

The mean QRS width decreased significantly after S/V therapy, reducing from 143.13 ± 13.7 to 125.4 ± 14.9 ms (*p* 0.000). Further classification based on QRS morphology revealed that the mean QRS duration decreased from 132.1 ± 14.3 to 123.6 ± 17.7 ms (*p* 0.000) among patients with LBBB morphology. In those with RBBB, mean QRS duration decreased significantly from 128 ± 13.7 to 120.3 ± 15.1 ms (*p* 0.01), and in patients with IVCD, mean QRS duration decreased significantly from 139.5 ± 11.8 to 130.3 ± 10.3 ms (*p* 0.000).

**Table 7:** Relationship between QRS morphology and QRS duration before and after S/V therapy

QRS morphology	QRS duration before S/V therapy Mean ± SD	QRS duration 6 months after S/V therapy Mean ± SD	Paired differences			*p* value
			Mean ± SD	95% Confidence interval of the difference		
				Lower	Upper	
LBBB	132.1 ± 14.3	123.6 ± 17.7	8.52 ± 8.3	5.11	11.9	0.000
RBBB	128 ± 13.7	120.3 ± 15.1	7.6 ± 10.3	2.13	13.1	0.01
IVCD	139.5 ± 11.8	130.3 ± 10.3	9.23 ± 6.9	6.4	12.1	0.000

LBBB, left bundle branch block; RBBB, right bundle branch block; IVCD, intraventricular conduction delay

## Discussion

This prospective study found that sacubitril and valsartan combination has an effect on ECG parameters such as mean QRS width and corrected QT interval, which were associated with the improvements in LV systolic function in HFrEF patients with idiopathic dilated cardiomyopathy.

The therapeutic benefits of the S/V combination have been well established, and its further effects on tissue remodeling are being investigated in various trials, including PRIME trial [18]. In fact, LV remodeling is an essential mechanism in the disease progression in HFrEF patients [19], and the PRIME study explored the potent additional reversal remodeling impact of sacubitril/valsartan [18]. This prospective randomized trial found that the S/V combination is more effective than ARBs in treating functional mitral regurgitation associated with heart failure. The authors discovered that S/V therapy reduces the regurgitated orifice area, left ventricular end-diastolic volume, left atrial volume, and the ratio of mitral inflow velocity to mitral annular relaxation velocity (E/E′) more than valsartan alone [18]. There was no benefit in LVEF, however, the authors excluded patients with LVEF ≤ 25% and individuals with substantial mitral regurgitation [18]. The prospective trial conducted by Bayard et al. [5] revealed that LVEF improved significantly (+ 3.6% in absolute value), which was most likely accounted by a large reduction in LVES volume.

The current study used a prospective design to evaluate the effect of S/V therapy on LV remodeling, and as stated in the results, LVEF significantly improved over 6 months after drug initiation, which is associated with a significant reduction in LVES and LVED dimensions, as well as a reduction in left atrial dimensions. However, there is no significant improvement in functional mitral regurgitationl7, which is most likely due to the small number of study individuals with severe mitral regurgitation.

Improved ventricular synchronization and electrical remodeling is substantially linked to favorable cardiac reverse remodeling. Kim et al. [20] investigated the potential effect of S/V therapy on QRS width in the ECG of HFrEF patients and discovered that changes in QRS width were significantly correlated to the changes in LVEF and LVESD, and these changes were a significant factor in predicting the recovery of LV systolic function and reverse cardiac remodeling. Our prospective investigation found that after S/V therapy, QRS width was significantly reduced, and this effect was significantly associated with LVEF improvement. In addition to QRS duration, there was a significant decrease in corrected QT interval, which was highly linked with LVEF improvement.

A few studies have looked into the impact of sacubitril/valsartan therapy on the QT interval in HFrEF patients. Gonçalves et al. [21] discovered that sacubitril/valsartan combination therapy reduced QRS duration and QTc interval significantly by 3.4% and 5.7%, respectively, as a consequence of the reverse remodeling process but this study only included 40 patients.

Several investigations have linked prolonged QT interval to particular echocardiographic features of LV remodeling, such as LV hypertrophy, left atrial dilatation, and decreased LV ejection fraction. According to the findings of Padmanabhan et al. [22], the presence of diastolic dysfunction (E/A ratio, DT) may indicate the presence of advanced LV dysfunction. As left ventricular disease progresses, myocardial stiffness increases and diastolic pressure rises. In patients with HFrEF, the increased LV filling pressure and subsequent subendocardial ischemia leads to conduction depolarization delay and QT prolongation. According to the current study, there is a significant improvement in corrected QT interval connected to improvement in LV systolic function, which may indicate its relationship to improvement in LV remodeling and the resulting improvement in repolarization and conduction delay. These results were similar to those of Okutucu S et al., who had 48 patients [22, 23].

Sub-analysis of the patients group with wide QRS complex > 120 ms, we found that improvement in mean QRS duration after S/V therapy regardless the type of conduction delay. QRS width significantly reduced in HFrEF patients with LBBB, RBBB, and IVCD. These findings support the beneficial effect of S/V treatment on cardiac reversal.

### Study limitations

The limited number of patients included in this study is a drawback, also it is only a single center study and we recommend large-scale multi-center investigations with a larger patient population.

## Conclusions

S/V therapy, in addition to improving echo parameters and NYHA class, improves QRS width and corrected QTc interval on ECG in HFrEF patients. This is an indication of reverse electrical LV remodeling and can be used as an auxiliary prediction for tracking therapy outcomes.

## Data Availability

They are available from the corresponding author on reasonable request.

## Abbreviations

ECG: Electrocardiography
ECHO: Echocardiography
RAAS: Renin angiotensin aldosterone system
ARNI: Angiotensin receptor neprilysin inhibitors
QTc: Corrected QT interval
SCD: Sudden cardiac death
EF: Ejection fraction
LA: Left atrium
LV: Left ventricle
HR: Heart rate
NYHA: New York heart association
ARB: Angiotensin II receptor blocker
ACEI: Angiotensin-converting enzyme inhibitors
BB: Beta blockers
MRA: Mineralocorticoid receptor antagonist
ICD: Implantable cardioverter defibrillator
IHD: Ischemic heart disease
MI: Myocardial infarction
CABG: Coronary artery bypass graft
PCI: Percutaneous coronary intervention
HFrEF: Heart failure with reduced ejection fraction
VHD: Valvular heart disease
DM: Diabetes mellitus
OSA: Obstructive sleep apnea
LBBB: Left bundle branch block
RBBB: Right bundle branch block
IVCD: Intraventricular conduction delay
